# Nomogram to predict symptomatic intracranial hemorrhage after mechanical thrombectomy in patients with intracranial atherosclerosis-related large-vessel occlusion

**DOI:** 10.3389/fneur.2026.1738518

**Published:** 2026-02-04

**Authors:** Hongchao Yang, Hongliang Zhong, Jianwen Jia, Yu Wang, Tong Li, He Liu, Yang Wang

**Affiliations:** Department of Neurosurgery, Beijing Chaoyang Hospital, Capital Medical University, Beijing, China

**Keywords:** endovascular treatment, intracranial atherosclerosis, large-vessel occlusion, nomogram, prediction, symptomatic intracranial hemorrhage

## Abstract

**Background:**

Despite significant advancements in endovascular treatment (EVT) over the past decade, symptomatic intracerebral hemorrhage (sICH) persists as a serious complication, especially in patients with acute ischemic stroke (AIS) related to intracranial atherosclerotic stenosis (ICAS), which is characterized by complex and calcified plaques. The present study aimed to develop a predictive nomogram for assessing the risk of sICH in AIS patients with ICAS and large-vessel occlusion (LVO) who undergo EVT.

**Methods:**

We analyzed data from 183 patients with acute ischemic stroke (AIS) due to intracranial atherosclerotic stenosis-related large vessel occlusion (ICAS-LVO) who underwent endovascular treatment (EVT) between January 2022 and December 2023. The primary safety outcome was the incidence of symptomatic intracranial hemorrhage (sICH). A nomogram was developed based on a multivariable logistic regression model, and the calibration of the model was evaluated using Spiegelhalter’s *Z*-test.

**Results:**

In our developed nomogram, elevated D-dimer levels (odds ratio [OR] 2.87; 95% confidence interval [CI], 1.53–5.39; *p* = 0.001), longer onset-to-recanalization time (OR 1.00; 95% CI, 1.00–1.01; *p* = 0.004), and larger core infarct volume (OR 1.04; 95% CI, 1.01–1.06; *p* = 0.002) were identified as independent predictors of symptomatic intracranial hemorrhage (sICH) in stroke patients undergoing endovascular treatment (EVT). The goodness-of-fit of the model was confirmed by a non-significant Spiegelhalter’s *Z*-test (*p* = 0.941).

**Conclusion:**

A nomogram integrating D-dimer levels, onset-to-recanalization time, and core infarct volume offers a reliable and practical method for estimating the risk of symptomatic intracranial hemorrhage in acute ischemic stroke patients with intracranial atherosclerotic stenosis-related large vessel occlusion receiving endovascular therapy.

## Introduction

Endovascular treatment (EVT) for patients with intracranial atherosclerotic stenosis-related large vessel occlusion (ICAS-LVO) presents unique technical challenges. The presence of complex and calcified atherosclerotic plaques often complicates the achievement of successful recanalization. Despite significant advancements in EVT over the past decade, symptomatic intracerebral hemorrhage (sICH) remains a serious complication that contributes to poor clinical outcomes (1, 2).

Intracerebral hemorrhage(ICH) location strongly influences both clinical manifestations, prognosis and etiology (3–5). Acute spontaneous lobar intracerebral hemorrhages present a more severe early prognosis compared to deep subcortical hemorrhages (5). Furthermore, the underlying causes exhibit location specificity, with lobar hemorrhages showing a higher association with non-hypertensive vascular pathologies than deep hemorrhages (5).

The complexity of ICAS lesions may increase the risk of sICH following mechanical thrombectomy (MT), making this issue a key concern in clinical practice. sICH is a devastating event, with randomized controlled trials (RCTs) reporting an incidence rate of approximately 4.4% (6). However, real-world data suggest that the incidence of sICH may be higher than that observed in clinical trials (7–10). Given these risks, identifying independent predictors of sICH is crucial for optimizing peri-procedural management in patients with LVO.

The aim of this study is to investigate the clinical and procedural factors associated with sICH in patients with ICAS-LVO undergoing EVT. A comprehensive understanding of these factors may facilitate the optimization of treatment strategies and enhance clinical decision-making when managing this complex patient population.

## Methods

### Population and data collection

We conducted a retrospective analysis using prospectively collected data from a comprehensive stroke center, covering the period from January 2022 to December 2023. Informed consent was obtained from patients or their proxies. Eligible participants were those diagnosed with ICAS-LVO stroke, with a time from onset to treatment of less than 24 h, aged 18 years or older, and having a baseline National Institutes of Health Stroke Scale (NIHSS) score of 5 or higher. Additionally, patients needed to have a premorbid modified Rankin Scale (mRS) score of 3 or lower. We excluded patients if their occlusion was not due to ICAS-LVO.

### Data collection

We collected baseline clinical data including demographics, medical history, NIHSS score, core infarct volume (measured using automated software), and time metrics such as time from symptom onset to groin puncture, time to recanalization, and total procedure duration. Other key data points included the site of occlusion, recanalization outcomes, occurrence of symptomatic intracranial hemorrhage (sICH), rescue treatment procedures, and clinical outcomes at 3 months. Two interventional neurologists independently assessed the etiology of the occlusion during the procedure, relying on patient history, risk factors, and angiographic features. Intracranial atherosclerotic disease(ICAD)-related occlusion was defined as either (1) > 70% fixed stenosis at the occlusion site or (2) mild stenosis with reduced blood flow or re-occlusion tendencies observed during the procedure or final angiography.

### Diagnosis of SICH

sICH was diagnosed according to the Heidelberg Bleeding Classification, based on the following criteria: (1) a total NIHSS score increase of ≥4 points compared to pre-worsening scores, (2) a ≥ 2 point increase in a single NIHSS category, (3) the need for intubation, hemicraniectomy, extraventricular drainage, or other significant interventions, (4) absence of alternative explanations for clinical deterioration, and (5) the presence of parenchymal hemorrhage type 2, even if the ischemic infarction contributed to the worsening condition.

### Outcome measures

Clinical prognosis was evaluated using the modified Rankin Scale (mRS) score at 3 months, with functional independence defined as an mRS score of 2 or lower. A favorable outcome was defined as an mRS score of 3 or lower. Reperfusion was measured using the modified Treatment in Cerebral Infarction (mTICI) scale, with successful reperfusion defined as a score of 2b, 2c, or 3.

### Statistical analysis

Continuous variables were expressed as medians with interquartile ranges (IQR 25–75) and compared using the Mann–Whitney U test where applicable. Categorical variables were presented as frequencies and percentages, and analyzed using either the chi-square test or Fisher’s exact test. We used LASSO (Least Absolute Shrinkage and Selection Operator) logistic regression to identify potential predictive factors, followed by multivariate logistic regression to determine significant predictors. Odds ratios (OR), 95% confidence intervals (CI), and *p*-values were reported. A nomogram was created to visually represent the predictive model, and model fit was assessed using Spiegelhalter’s Z-test. Finally, decision curve analysis (DCA) was performed to evaluate the model’s clinical utility. All statistical analyses were performed using R software (Version 4.4.1,^1^) with a significance level of *p* < 0.05.

## Results

### Demographic and clinical characteristics

A total of 183 patients with acute ischemic stroke (AIS) were included in the study. Among them, 22 patients developed sICH, while 161 did not. Table 1 presents the demographic, clinical, and laboratory characteristics of patients grouped by sICH and non-sICH. Patients who developed sICH were older (67 vs. 64 years old), had significantly higher D-Dimer levels (1.18 vs. 0.82), higher NIHSS scores at admission (22 vs. 15), larger infarct core volumes (21.9 mL vs. 10.0 mL), longer onset-to-recanalization times (713 min vs. 510 min), and a greater proportion of unfavorable outcomes (mRS 3–6) (81.8% vs. 41.0%).

**Table 1:** Characteristics of all patients.

Variables	sICH group (*n* = 22)	Non-sICH group (*n* = 161)	*p*
Age (years, median, IQR)	67.0 (64.8–73.3)	64.0 (56.5–71.0)	0.017
Sex (*n*/%)			1.0
Male	18 (81.8)	133 (82.6)	
Female	4 (18.2)	28 (17.4)	
History of hypertension (*n*/%)	12 (54.5)	67 (41.6)	0.262
History of diabetes (*n*/%)	6 (27.3)	23 (14.3)	0.126
History of coronary heart disease (*n*/%)	4 (18.2)	11 (6.8)	0.088
History of atrial fibrillation (*n*/%)	1(4.5)	2 (1.2)	0.321
History of cerebrovascular disease (*n*/%)	3 (13.6)	19 (11.8)	0.733
History of smoking history	12 (54.5)	82 (50.9)	0.750
TT (median, IQR)	16.1 (15.7–16.5)	16.5 (15.8–17.7)	0.187
PT (median, IQR)	12.3 (11.7–12.7)	12.7 (11.8–13.1)	0.082
INR (median, IQR)	1.04 (1.01–1.09)	1.01 (1.00–1.02)	0.298
APTT (median, IQR)	29.8 (26.9–38.8)	27.2 (25.6–32.4)	0.597
FIB (median, IQR)	288.8 (266.8–483.9)	294.3 (244.8–373.7)	0.359
D-Dimer (median, IQR)	1.18 (0.83–3.82)	0.82 (0.45–1.52)	<0.001
WBC (median, IQR)	9.05 (7.62–13.45)	7.96 (7.38–11.09)	0.270
PLT (median, IQR)	188.0 (156.0–222.0)	203.0 (179.0–234.0)	0.543
IV tPA (*n*/%)	2 (9.1)	9 (5.6)	0.625
NHISS score (median, IQR)	22 (15–26)	15 (10–22)	0.003
Core infarcted volume (mL, median, IQR)	21.9 (10–71.3)	10.0 (0–30.0)	<0.001
Onset-to-recanalisation time (min, median, IQR)	713.0 (581.3–839.3)	510.0 (360.0–751.5)	0.012
Occlusion site (*n*/%)			0.798
ICA	12 (54.5)	77 (47.8)	
MCA	5 (22.7)	48 (29.8)	
VA-BA	5 (22.7)	36 (22.4)	
mTICI (*n*/%)			1.0
0-2a	2 (9.1)	17 (10.6)	
2b-3	20 (90.6)	144 (89.4)	
Rescue treatment (*n*/%)			0.949
Tirofiban	5 (22.7)	33 (20.5)	
Balloon angioplasty	4 (18.2)	28 (17.4)	
Stent angioplasty	11 (50.0)	100 (62.1)	
mRS score of 3 months after operation (*n*/%)			<0.001
0–2	4 (18.2)	95 (59.0)	
3–6	18 (81.8)	66 (41.0)	

IQR, Interquartile range; NIHSS, National institutes of health stroke scale; TT, Thrombin time; PT, Prothrombin time; INR, International normalized ratio; FIB, Fibrinogen; APTT, Activated partial thromboplastin time; WBC, White blood cells; PLT, Platelets. IA, intra-arterial; ICA, internal carotid artery; ICH, intracranial hemorrhage; INR, international normalized ratio; IV tPA, intravenous tissue plasminogen activator; MCA, middle cerebral artery; NIHSS, national institutes of health stroke scale.

Among the 22 patients with sICH, IVH was observed in 11 cases (50.0%). At the 3-month follow-up, poor functional outcome (mRS score 4–6) was observed in 9 of these 11 patients with IVH (81.8%). In contrast, among the 11 patients without IVH, 4 (36.3%) had a poor outcome. Prognosis did not differ significantly among these groups (*p* = 0.091).

AS for sICH location, we observed 27.3% (6/22) cases of lobar, 36.4%(8/22) of deep subcortical, and 36.4%(8/22) of combined (both locations) hemorrhage. No significant difference in prognosis was observed among the groups (*p* = 0.450).

A total of 27 patients (14.7%) died during the study. Mortality was higher in the sICH group (8/22, 36.4%) than in the non-sICH group (19/161, 11.8%). In the sICH group, 4 in-hospital deaths were all due to cerebral herniation. Among the 4 deaths during the 3-month follow-up, causes included pneumonia (*n* = 2), cardiac disease (*n* = 1), and malignant cerebral edema secondary to recurrent infarction (n = 1). In the non-sICH group, there were 3 in-hospital deaths from heart failure and 16 deaths during follow-up, primarily from pneumonia (*n* = 9) and cardiac disease (*n* = 7).

Multivariable logistic regression analysis identified D-Dimer (OR 2.87, 95% CI 1.53–5.39, *p* = 0.001), onset-to-recanalization time (OR 1.00, 95% CI 1.00–1.01, *p* = 0.004), and core infarct volume (OR 1.04, 95% CI 1.01–1.06, *p* = 0.002) as independent predictors of sICH (Table 2). The multivariate analysis demonstrated a positive predictive value of 83.3%, a negative predictive value of 95.7%, and a diagnostic accuracy of 94.5%.

**Table 2:** Multivariable logistic regression for unfavorable prognosis.

Variables	OR	95% CI	*p*-value
D—dimer	2.87	1.53–5.39	0.001
Onset-to-recanalisation time	1.00	1.00–1.01	0.004
Core infarcted volume	1.04	1.01–1.06	0.002

OR, Odds Ratio; CI, Confidence Interval.

### Identification of significant predictors for sICH following EVT

**Figure 1:**
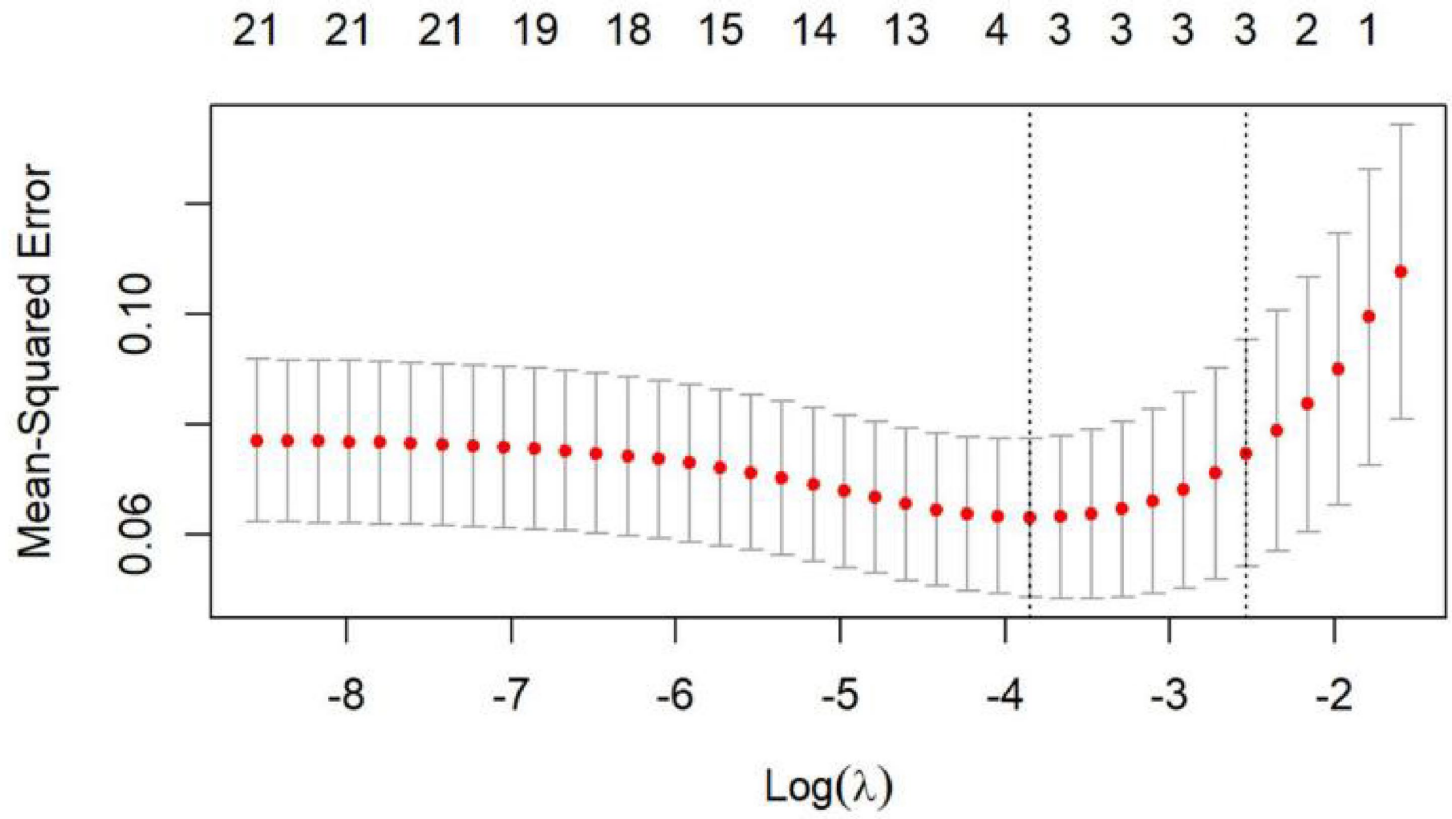
Factor selection using the least absolute shrinkage and selection operator (LASSO) logistic regression. The LASSO coefficient profiles of the 22 candidate variables. The LASSO regression analysis.

To identify the key predictors of unfavorable outcomes following EVT, the LASSO logistic regression algorithm was applied. This method selected the most significant predictive factors from the variables listed in Table 1. Twenty-two factors with non-zero coefficients were selected based on the optimal lambda value (Figure 1). Finally, eight factors were included in the final model: thrombin time, fibrinogen, white blood cell count, age, D-Dimer, onset-to-recanalization time, core infarct volume, and NIHSS score at admission. These factors were subsequently analyzed through multivariable regression.

**Figure 2:**
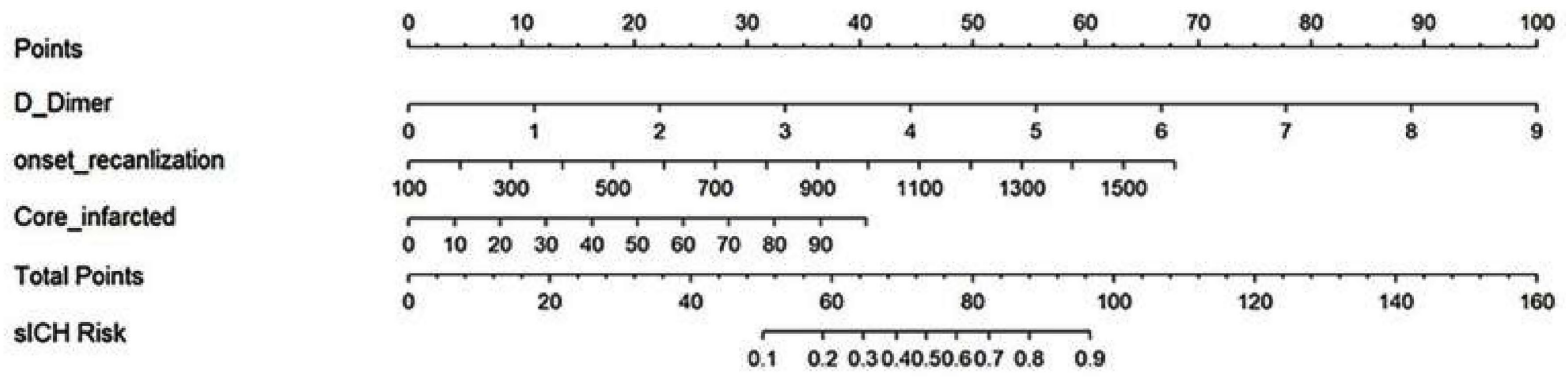
Nomogram for predicting the probability of symptomatic intracranial hemorrhage after mechanical thrombectomy.

We then constructed a nomogram model to estimate the likelihood of sICH. Each independent predictor was assigned a score between 0 and 100, with the total score representing the overall probability of developing sICH. The total possible score ranged from 0 to 130 points (Figure 2).

### Performance and validation of the nomogram

**Figure 3:**
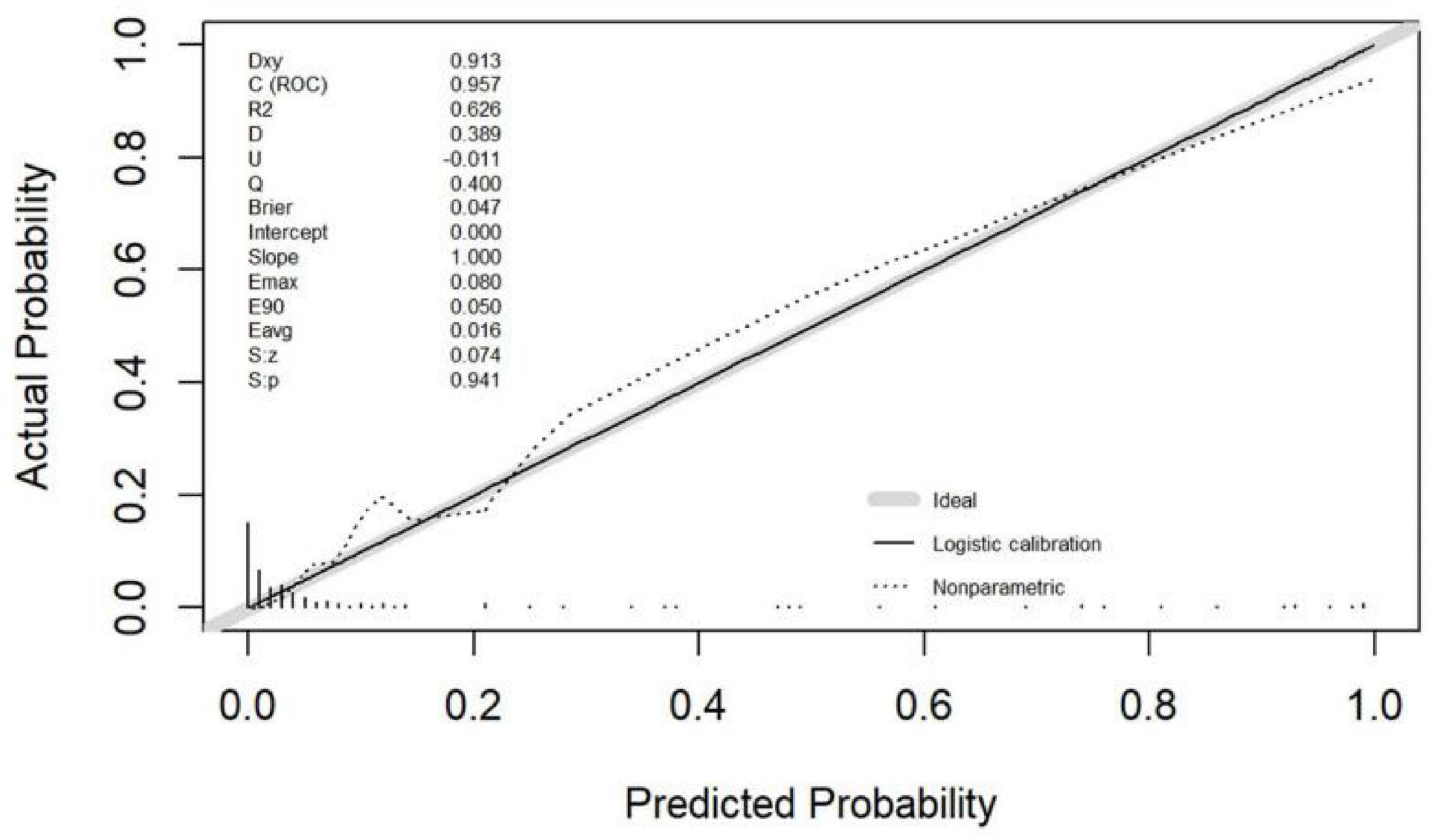
The calibration plot of the nomogram for symptomatic intracranial hemorrhage of stroke patients undergoing mechanical thrombectomy.

**Figure 4:**
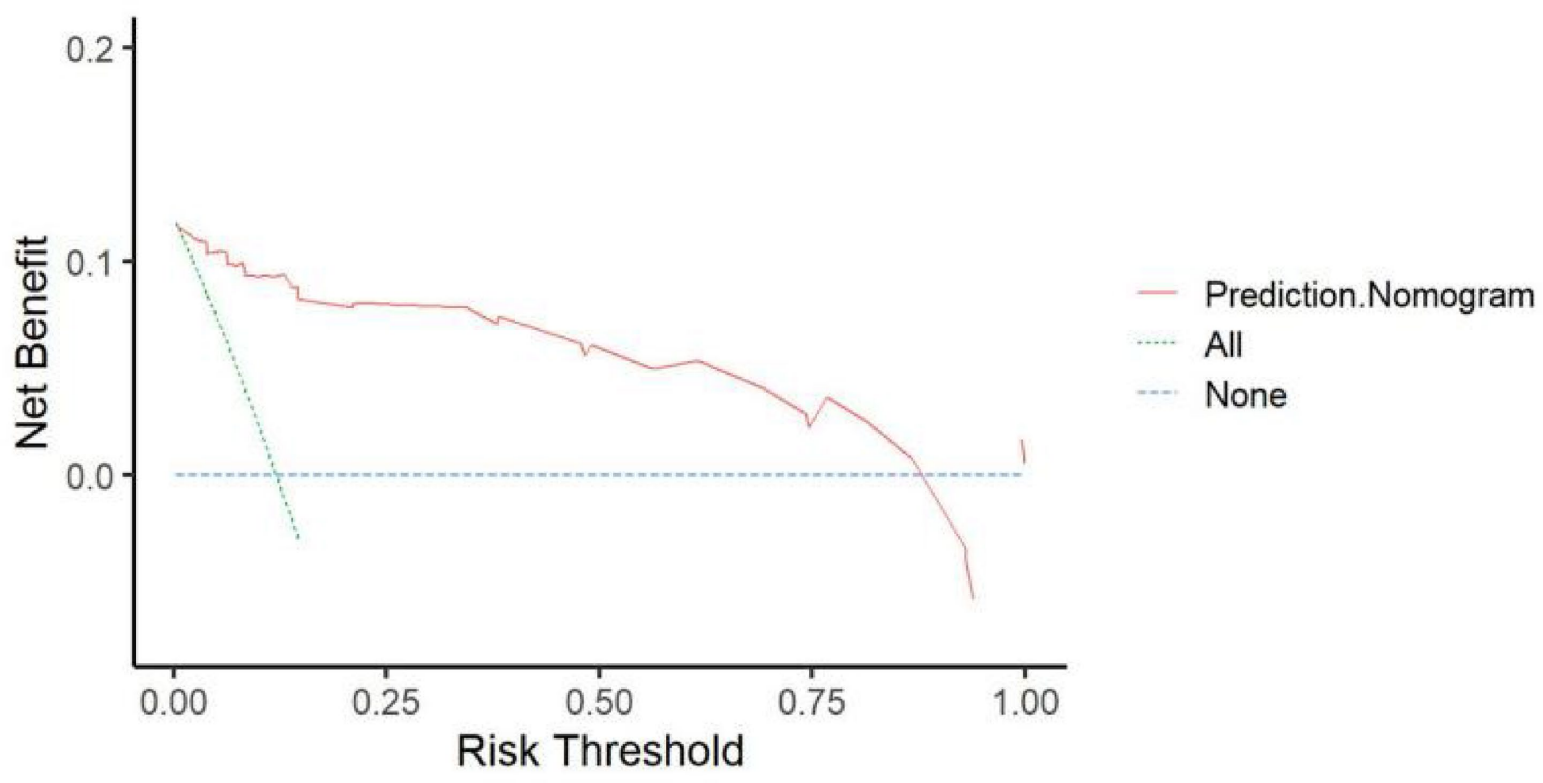
Decision curve analysis (DCA) of the nomogram. *x*-axis, the threshold probability; *y*-axis, the net benefit.

The calibration curve for the nomogram predicting unfavorable prognosis in stroke patients showed strong agreement with actual outcomes (Figure 3). The Spiegelhalter’s Z-test indicated no significant deviation from a perfect fit (*p* = 0.941), and the Brier score was 0.047, indicating that the nomogram closely aligned with the ideal predictive model. Figure 4 demonstrates that when the risk threshold ranged from 5 to 90%, the nomogram offered a net benefit in predicting sICH after mechanical thrombectomy (MT) compared to treating all or none of the AIS patients.

## Discussion

In this retrospective study, we developed a nomogram incorporating onset-to-recanalization time, core infarct volume, and D-Dimer levels to predict the risk of symptomatic intracranial hemorrhage (sICH) in patients with ICAS-LVO following endovascular treatment (EVT). The clinical utility of this model was validated through decision curve analysis (DCA), which demonstrated significant net benefit within specific probability ranges. Early prediction of sICH is crucial for optimizing EVT in ICAS-LVO patients, and this nomogram provides a valuable tool for clinicians to guide treatment decisions and improve patient outcomes.

Previous studies have shown that the incidence of sICH after thrombectomy is higher in real-world settings than in randomized controlled trials (RCTs) (7–10). This is particularly true for patients with ICAS-LVO, where the complexity of ICAS lesions often poses challenges for neurointerventionalists, resulting in longer procedure durations (11, 12). Our study confirmed that longer onset-to-recanalization times are a significant predictor of sICH. This reflects the technical difficulties encountered during EVT for ICAS, as more attempts are often required to achieve successful recanalization. Our findings align with those of Kass-Hout et al. (13) who reported that longer procedure times are independently associated with sICH in patients undergoing thrombectomy for LVO stroke. To minimize the risk of sICH, it is essential to select the most appropriate techniques and devices to reduce procedure duration while achieving effective recanalization.

In our study, baseline infarct core volume also emerged as an independent predictor of sICH. This result is consistent with recent studies that reported an increased risk of sICH in patients with large infarct cores despite the benefits of EVT (14–16). Prior to these RCTs, studies using the ASPECTS score to measure infarct core found that a low ASPECTS score was a predictor of sICH after EVT (7, 17, 18). Several factors may explain why larger infarct cores increase the risk of sICH: reperfusion syndrome due to the rupture of necrotic vessel walls, and increased blood–brain barrier permeability caused by large infarct and edema volumes (19). These conditions are more likely to occur in patients with larger infarct cores, increasing the risk of complications like sICH, which can worsen clinical outcomes. Therefore, the risk–benefit ratio of thrombectomy must be carefully considered in patients with larger infarct cores.

Interestingly, we also found that D-Dimer levels were an independent predictor of sICH. D-Dimer is present in negligible amounts in healthy individuals but rises significantly in patients experiencing thrombotic or hyperfibrinolytic activity (20, 21). Previous studies have linked high D-Dimer levels with thrombotic diseases and stroke (22). Its dual role in hypercoagulation and subsequent fibrinolysis may contribute to hemorrhagic transformation in AIS patients after thrombectomy. Elevated baseline D-Dimer levels may indicate ongoing microthrombosis in the microcirculation of the occluded vessel, leading to extensive activation of the coagulation system, hyperfibrinolysis, and coagulopathic disturbances (23). This finding suggests that D-Dimer levels could be useful for early prediction of hemorrhagic transformation, and may aid in perioperative management by informing decisions on thrombectomy attempts, procedure timing, and the use of antithrombotic or anticoagulant therapies.

Multiple studies have established that the presence of intraventricular hemorrhage (IVH) is independently associated with increased mortality and poorer functional outcomes (24–26). Furthermore, IVH is closely linked to hematoma expansion (HE), a well-recognized predictor of unfavorable prognosis. Evidence also indicates that the detection of IVH on follow-up CT scans independently correlates with early hematoma expansion (27). In our study, patients with IVH extension exhibited a trend of poorer prognosis, which is consistent with previous research (24–26). Future studies could conduct more in-depth analyses by incorporating factors such as IVH severity scores.

This study has several limitations. First, it was a single-center, retrospective study with a relatively small sample size, which limits the generalizability of the findings to other populations and settings. A multicenter, prospective trial is necessary to validate the accuracy of the model. Second, not all potential risk factors were included in the analysis, meaning that additional predictors could enhance the model. Future studies should explore other parameters and investigate whether integrating additional biomarkers could improve the nomogram’s accuracy. Finally, while our study used prospectively collected data and the model showed good discriminative performance, external validation is needed to further assess its robustness and applicability in broader clinical settings.

Future research could integrate multi-omics data—such as genomic, proteomic, or metabolomic profiling of patients—to identify specific biomarkers associated with the occurrence of sICH. Furthermore, in-depth exploration of the underlying pathophysiological mechanisms and differentiation among hemorrhage subtypes may help develop more targeted prevention and management strategies tailored to sICH resulting from distinct etiological pathways.

## Conclusion

A novel nomogram incorporating onset-to-recanalization time, core infarct volume, and D-dimer levels has demonstrated the ability to predict sICH in AIS patients with ICAS-LVO treated with EVT. Larger prospective studies are warranted to further validate the effectiveness of this predictive tool.

## Data Availability

The datasets presented in this article are not publicly available because they are intended for academic research only. Requests to access the datasets should be directed to HY, yhchao2006@163.com.

## Footnotes

1
https://www.R-project.org
